# Supplementary material for: Type B CTD Proteins Secreted by the Type IX Secretion System Associate with PorP-like Proteins for Cell Surface Anchorage
Source: Int J Mol Sci. 2022 May 19;23(10):5681. doi: 10.3390/ijms23105681 (PMC9143113; doi:10.3390/ijms23105681)
Supplement: Supplementary file 1 [file ijms-23-05681-s001.zip › ijms-1722791-supplementary.pdf]

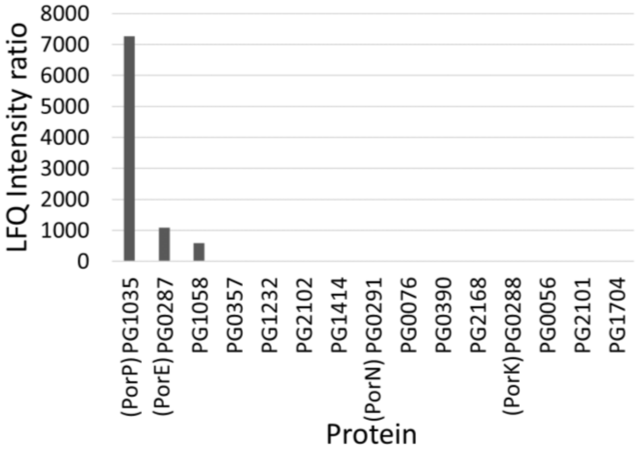

Supplementary Figure 1: PG1035-PorP-PorE-PorK-PorN complex

P. gingivalis wildtype and the *pg1035* mutant strain were subjected to co-immunoprecipitation with agarose beads bound to PG1035 antibodies. The immunoprecipitated samples were digested with trypsin and analysed by mass spectrometry and quantified using MaxQuant software. The ratio of LFQ intensities of WT to *pg1035* mutant was plotted.
